# Effects of singing on vascular health in older adults with coronary artery disease: a randomized, crossover trial

**DOI:** 10.3389/fcvm.2025.1546462

**Published:** 2025-04-28

**Authors:** Mehri Bagherimohamadipour, Muhammad Hammad, Alexis Visotcky, Rodney Sparapani, Jacquelyn Kulinski

**Affiliations:** ^1^ Division of Cardiovascular Medicine, Medical College of Wisconsin, Milwaukee, WI, United States; ^2^ Division of Biostatistics, Data Science Institute, Medical College of Wisconsin, Milwaukee, WI, United States

**Keywords:** cardiac rehabilitation, endothelial function, vascular function, heart rate variability, singing

## Abstract

**Background:**

The impact of singing on cardiovascular health has not been extensively studied. The aim of this study is to investigate the effect of singing on cardiovascular biomarkers in an aging population with coronary artery disease (CAD).

**Methods:**

Participants had three study visits separated by 2–7 days, according to a randomized, researcher-blinded, crossover, controlled design: (1) a 30-min period of live singing with an in-person music therapist, (2) a 30-min period of singing along to an instructional video and (3) a 30-min rest (control). Primary outcomes included macrovascular endothelial function assessed by brachial artery flow-mediated dilation and microvascular function assessed by peripheral arterial tonometry [Framingham reactive hyperemia index (fRHI) and reactive hyperemia index (RHI)]. Heart rate variability (HRV) was a secondary outcome.

**Results:**

Sixty-five subjects (mean age 67.7 ± 0.8 years, 40% female) completed the study. Compared to control, there was an increase in fRHI for the singing video intervention (estimate: 0.54, SE: 0.19, *p* = 0.005) but not for the live singing intervention (estimate: 0.11, SE: 0.18, *p* = 0.570). There was no change in macrovascular function with either intervention. The low frequency/high frequency (LF/HF) ratio increased by 2.80 (SE: 1.03, *p* = 0.008), and the natural logarithm of high frequency (LnHF) power decreased by −0.90 ms^2^ (SE: 0.29, *p* = 0.003) with the video (during to pre-change). When assessing post- to pre- change, the live singing intervention showed a significant change of −0.62 ms^2^ (SE 0.29, *p* = 0.036) in LnHF power.

**Conclusions:**

Singing along to an instructional video for 30 min improved microvascular, but not macrovascular, endothelial function, in older patients with CAD. HRV changes with singing are similar to that of exercise.

**Clinical trial registration:**

ClinicalTrials.gov, identifier (NCT04121741).

## Introduction

Exercise promotes healthy vascular aging and is a crucial means for prevention, treatment, and rehabilitation of cardiovascular disease (CVD). According to data from the 2020 National Health Interview Survey in the U.S., fewer than one-fourth (24.2%) of adults meet national recommendations for physical activity (at least 150 min of moderate-intensity exercise per week) (1). However, this percentage is markedly lower for adults ages 65 and over, with only 15.3% of males and just 10.8% of females meeting national recommendations for physical activity (1). Similarly, older adults are less likely to participate in cardiac rehabilitation (CR) programs, even when referred (2–6). CVD in older adults is usually complicated by age-related complexities, including multi-morbidity, polypharmacy, frailty, deconditioning, falls, disability, and other challenges that make participation (or the perception of participation) in CR difficult (4, 7). Alternative forms of therapy to reduce CVD burden and improve health are needed in this aging population.

Music as a therapeutic is attractive for a variety of reasons including minimal risk to patients, ease of use, accessibility, and pervasiveness across culture. Singing is increasingly being recognized for its positive effects on overall mental and physical health (8). Group singing interventions have been suggested to improve health-related quality of life, as well as reduce anxiety and depression (9). Singing can cause changes in neurotransmitters and hormones, including the upregulation of oxytocin, immunoglobulin A, and endorphins, which improves immune function and increases feelings of happiness (8, 10). Singing has also shown promise in improving lung function in patients with chronic obstructive pulmonary disease and asthma (11). A single-center randomized trial of 24 subjects with structural heart disease demonstrated an improvement in respiratory muscle strength and quality of life after 12 weeks of weekly choir rehearsal and daily breathing exercises, compared to standard care alone (12). Despite promising findings on the various outcomes in other chronic conditions, only a limited number of studies have evaluated the effects of singing on cardiovascular health, and to our knowledge, none have enrolled subjects with established coronary artery disease (CAD) (13). In a narrative review article that included 26 intervention studies and impacts on cardiovascular health, only two studies examined singing (compared to music listening) (13).

Different types of singing can produce varying results, as demonstrated by Vickhoff et al, where 5 min of mantra singing in a choir led to the most significant increase in root mean square of successive differences (RMSSD) compared to hymn singing or humming in 15 young, healthy adults (14). A study has suggested that reciting Rosary and mantra (meditative vocal production such as chanting) can positively affect the cardiovascular system by enhancing heart rate variability (HRV) (15). In a crossover study of 20 young healthy adults, Bernardi et al. compared the effects of 7 min of toning (improvisation-based open vowel vocalization), singing and matched silent breathing conditions on cardiorespiratory physiology. While HRV, measured as the standard deviation of NN intervals (SDNN), increased in all conditions compared to baseline, it was significantly higher during toning, which induced a breathing frequency of 0.1 Hz, compared to singing (16). Five additional studies found physiological synchronization between individuals while singing in a choir in unison, specifically in breathing and HRV (17–21). One possible explanation for these findings could be that different types of singing have varying effects on respiration rates. A slower breathing rate of 0.1 Hz has been shown to enhance the effects of respiratory sinus arrhythmia (RSA), a phenomenon describing the coupling of HRV to respiration (22). Furthermore, the physiological demands of singing are comparable with walking at a moderately brisk pace (23), providing biological plausibility that the health benefits with singing may overlap with that of exercise. However, unlike traditional physical exercise, the impact of singing on cardiovascular health in adults with CAD has not been extensively studied.

Vascular endothelial function strongly predicts cardiovascular events in patients with and without CVD (24), and endothelial dysfunction can be reversed by interventions (such as physical exercise) known to reduce CVD risk (25, 26). Our team has been the first to demonstrate that just 14 min of solo singing improves endothelial function acutely, regardless of singing expertise (27). Subjects watched and sang along to a 14-min instructional video created by a music professor, which included vocal warm-up exercises followed by the Star-Spangled Banner, repeated at various tempos and pitches. During this video, the professor played the piano and coached the subject through the warm-up and singing. The warm-up included humming, semi-occluded vocal tract exercises, and toning. Lyrics were displayed along the bottom of the video. Changes were more robust in subjects with baseline endothelial dysfunction (such as those with CAD). Major limitations of the prior study included lack of randomization and a control.

HRV is the variability between R-R intervals in successive heartbeats and is the result of a complex interaction between respiratory activity and autonomic cardiovascular control between the two branches of the autonomic nervous system (sympathetic and parasympathetic) (28). Measurements of HRV have been found to be powerful predictors of cardiac morbidity and mortality (29, 30). A meta-analysis by Jarczok et al. that included over 38,000 participants showed that lower SDNN and low frequency (LF) power were significantly associated with higher cardiac and all-cause mortality, regardless of whether the model was unadjusted or covariate adjusted. In addition, RMSSD and high frequency (HF) power showed the strongest association with all-cause mortality in unadjusted models (31). While singing has been shown to improve HRV in young, healthy populations (14), there is no data on the impact of singing on HRV in patients with CVD. This is a secondary outcome measure that we plan to explore here.

The pilot study conducted by our team found that singing along to a pre-recorded video improved peripheral (micro)vascular endothelial function (27). Research studies involving music therapy interventions (delivered by board-certified music therapists) led to more consistent and positive results across studies compared to music listening studies (music listening to pre-recorded video and music- guided relaxation), likely due to the specialized training to meet the person's in-the-moment needs during live music (32). Because we hypothesize that some of the benefits of physical exercise may extend to the physical activity of singing, we considered that incorporating a music therapist (to increase level of exertion) might be more impactful.

The aim of this study is to investigate the impact of two different singing interventions on important cardiovascular biomarkers, including vascular endothelial function and HRV, in an aging population with established CAD. We assess macrovascular endothelial function using the brachial artery flow-mediated dilation (FMD) and the microvascular endothelial function using peripheral arterial tonometry (PAT) to measure Framingham reactive hyperemia index (fRHI) and reactive hyperemia index (RHI). We hypothesize that older patients with established CAD will show an improvement in these cardiovascular biomarkers after 30 min of singing.

## Methods

### Subject recruitment

All subject-related study activities were performed at a large academic medical center after approval by the local Institutional Review Board (IRB). Subjects were identified by reviewing the electronic medical records of patients visiting the outpatient cardiology clinics. To increase enrollment of females, as well as black, and Hispanic patients, of both genders, potential subjects were also identified using a cohort discovery tool (TriNetX) from the institution's Clinical Research Data Warehouse. The Honest Broker tool was used to extract desired patient information including patient demographics, medication history, diagnosis/problem list, provider notes and lab/imaging results for each identified subject.

The eligibility criteria for the study included patients between 55 and 79 years of age with a history of CAD defined as having a history of myocardial infarction (MI), coronary artery stenosis greater than 50%, percutaneous coronary intervention with stent placement and/or balloon angioplasty, or coronary artery bypass grafting (CABG). We excluded patients who had a permanent pacemaker or implantable cardioverter defibrillator (ICD), history of atrial fibrillation, atrial flutter or atrial tachycardia, Parkinson's disease or a tremor, amputated upper extremity or presence of upper-arm (dialysis) fistula, fingernail onychomycosis (fungal infections resulting in thickening of the nails), pregnancy, current illicit drug use (marijuana, tobacco, cocaine, amphetamines, etc.), current excessive alcohol use (defined as more than 14 drinks/week for females, more than 28 drinks/week for males), unstable CAD (active symptoms of chest discomfort), history of a stroke or transient ischemic attack (TIA), peripheral arterial disease, known history of cognitive impairment or inability to follow study procedures, cancer requiring systemic treatment within five years of enrollment, subjects requiring supplemental oxygen use, dosing changes of vasoactive medications in the 6 weeks prior to enrollment, and non-English speaking subjects (instructional videos with lyrics were recorded in English).

### General study design

Eligible patients meeting the inclusion and exclusion criteria were contacted via phone or email to provide a brief overview of the study, to assess their interest in the study and to determine initial eligibility in the form of a short screening questionnaire. A detailed screening visit was then completed via a phone call to review the informed consent, the patient's medical history, and to schedule their three in-person study visits. The medical history form included demographics, medical history, current medications, and physical limitations and/or mobility issues.

At the first study visit, a written informed consent was obtained prior to initiating any study related activities, and the subject underwent randomization in a researcher-blinded, control, crossover design fashion with the order of the three study visits randomized. Participants each had three visits on three different occasions for the following interventions: (1) a 30-min period of live singing with an in-person, board-certified music therapist, (2) a 30-min period of singing along to an instructional video and (3) a 30-min rest (control) visit. The advantage of the crossover design is that each subject serves as his or her own control, significantly reducing between-subject variability, and allowing the detection of smaller effect sizes with reduced sample sizes. The design can also reduce the chance of confounding bias by evaluating all participants for all treatments (33). The participants were randomized, stratified by gender, using an electronic randomization scheme created by an independent statistician not associated with this study. Patients were asked to come to all three study visits fasting from food or beverage (other than water) for 8 h prior to the visit and were asked to hold their morning medications for the vascular function measurements (all study visits were conducted in the morning). Baseline vital signs (heart rate, blood pressure, pulse oximetry), weight and height, and baseline measurements of HRV, brachial artery FMD, and PAT, were taken prior to the intervention.

To ensure rigor of the study, all study personnel including the principal investigator, biostatisticians, sonographers, and the voice professor were kept blinded to the intervention. The only two people unblinded to the intervention were the research coordinator, who scheduled and conducted the study visits, and the music therapist, who led the live singing sessions. The music therapist and research coordinator were not involved in data analysis. A clinical trials compliance coordinator was assigned to this study to ensure protocol compliance and consistent delivery of singing and control interventions.

### Singing protocols

To improve the transparency and specificity of reporting music-based interventions, a set of specific reporting guidelines for music-based interventions was followed (34). The subjects attended two different singing interventions visits and one control visit (rest period), each 30 min in duration, in three separate visits according to randomized, controlled, researcher-blinded, crossover design. The study visits took place in a private research suite with very little ambient noise. All interventions were conducted with the subject in a seated position. There was a smaller adjoining room where research staff sat in the background, affording some privacy to the participant during singing interventions. The visits were separated by a minimum of 2 days (up to 7 days) to allow for potential wash-out effects of prior intervention visits. The minimal duration of 2 days was selected based on prior research demonstrating normalization of the FMD response at 24–48 h after an acute bout of exercise (35). As a result of the COVID-19 pandemic, 20 out of 64 live music sessions were conducted by the music therapist remotely.

A video series was specifically created and recorded for the purposes of this study. The video singing intervention included an instructional sing-along video displaying a voice professor playing the piano and directing an elderly student in singing. Using her own BRAVE (Bringing Awareness to Vocal Exploration) Work System, the 10-min vocal warm-up video was devised by the voice professor to build awareness in the singer within the four main pillars of the system-Breath/Body, Vocal Tract, Face, and Tongue. The session begins with simple awareness of being seated in the chair and continues to add layers of awareness with intentional Breathing. Semi-Occluded Vocal Tract (SOVT) activities are added to each exhale, using [s], [z], [v], lip trills (horse lips) reflexive [r] and humming, each offering a bit of interoception of the Vocal Tract in action. In each case, the singer would be encouraged to modulate the pitches, creating a siren, by continuing to “speak through” each sound. Subjects were encouraged to decrease or increase the pitch to their comfort levels. Next, activities designed to “wake up” the Face using a 5-note, descending scale were demonstrated, for example “Mona Lisa bites an apple and says ‘WOW’.” The slight smile of the Mona Lisa as she opens her mouth to bite an apple is a helpful image to allow for appropriate engagement of the facial muscles involved in singing. A short Tongue sequence designed to stretch the tongue root is demonstrated. Finally, the tongue is brought in to the “bite the apple” face with ‘ay-ay-ay-ay-ay’ and ‘why-oh-why-oh-why’ using the same 5-note scale pattern. This brings it all together and completes the 10-min warm-up. The subjects selected 2 songs to sing for 10 min each from four different music genres including Folk (*This Land is Your Land)*, Pop *(Hey Jude)*, Country *(Jolene)*, and Hymn *(Amazing Grace)*—varying in tempo, melodic contour, and rhythm to fill the full 10 min per song. Lyrics were displayed along the bottom of the video screen on a Microsoft surface laptop. Volume was set on maximum (Realtek Audio).

The live music intervention included a live in-person singing session with a board-certified music therapist, who alternated between the keyboard or guitar depending on the subjects' music selection. This session also included the 10-min vocal warm-up followed by two songs at 10 min each. Song choices were selected by the subject from a multi-genre binder targeting adults ages 55–79 and including 40 song choices (Supplementary Table 1). The music therapist encouraged more respiratory exertion since research shows that higher levels of respiratory exertion, such as done during physical exercise, result in improved cardiovascular health (36). Prior to the live sessions, the music therapist would address any apprehensions subjects may have about singing in front of others while emphasizing that it does not matter how they sound but rather, how they feel. During the live sessions with the music therapist, increased participation was encouraged through non-verbal cues, such as leaning in towards the participant, nodding of the head to indicate they were doing well and on track. In-between songs, the music therapist would provide verbal praise and encouragement and validate that the changes in tempo may be unfamiliar to them. She encouraged them to have fun and simply follow along with her.

During the control intervention, subjects had a 30-min period of rest, sitting upright as they would be for the singing intervention. During this rest period, they were not allowed to sleep, watch television, read, browse their smartphone, or listen to music. During this visit, study subjects underwent a validated, tablet-based screening test for hearing loss (37).

### Measures of vascular endothelial function

Vascular function measurements were performed before and after each singing and rest intervention with subjects in the supine position in a hospital bed for at least 20 min, in a quiet, temperature-controlled room (70–75 °F) with a non-condensing humidity. The brachial artery FMD technique (24) was used to assess macrovascular function, and PAT (38, 39) was used to assess microvascular function. FMD is a non-invasive measure of vascular endothelial function that uses high-resolution ultrasound technology (7.5–13 Hz probe) to measure changes in brachial artery diameter in response to reactive hyperemia after a 5-min period of arterial occlusion (>200 mm Hg systolic or at least 60 mm Hg above resting systolic blood pressure with the blood pressure cuff below the elbow). On rapid deflation of the cuff, transient hyperemia stimulates nitric oxide production and release from the endothelium, resulting in dilation. The protocol has been previously described in detail (40). FMD is expressed as the change in post-stimulus diameter as a percentage of the baseline diameter (FMD%). Because there is a high degree of technical skill related to assessing FMD, we routinely assess the reproducibility of these measurements in our lab and have shown excellent reproducibility across technicians (41).

Microvascular endothelial function by digital PAT (Endo-PAT 2000, Itamar Medical, Israel) is expressed as RHI and fRHI (38, 42). Endothelium-mediated changes in vascular tone after occlusion of the brachial artery reflect a downstream hyperemic response (43). Disposable, pneumatic finger probes were placed on both index fingers. Each recording included 5 min of baseline measurement, 5 min of occlusion, and 5 min post-occlusion (hyperemic period). PAT measurements were taken simultaneously with FMD.

We used a nitroglycerin mediated dilation (NMD) test to differentiate between endothelium-dependent vasodilation and endothelium-independent vasodilation (44). Measurement of blood vessel diameter was done before and after the subject was given a pill of nitroglycerin (0.4 mg) sublingually (no blood pressure cuff inflation). NMD was only done once during each study visit, after the second FMD and PAT measurements were completed. NMD was optional and was not given if the participant met at least one of the following criteria: systolic blood pressure less than 100 mmHg; prior intolerance or adverse reaction to nitroglycerin; history of migraine headaches; or sildenafil, tadalafil, or vardenafil use within a week of participating in the study.

### Heart rate variability (HRV)

An appropriately sized (Bluetooth-capable) Polar H7 chest strap (Polar, Kempele, Finland) with a heart rate sensor was applied to the subject's bare chest. Polar H7 uses a one lead electrocardiogram (ECG) with two electrodes to measure heart rate at a sampling frequency of 1,000 Hz. Three-minute-long HRV recordings were obtained before, during (20–25 min in), and after singing (or rest control). This duration of HRV recording has been shown to be accurate at rest and post-exercise (45). The data was transmitted to an iPad and analyzed using the Elite HRV application (Asheville, NC). The normal-to-normal (NN) intervals included all intervals between adjacent QRS complexes resulting from sinus node depolarizations. HRV (time domain) was reported as SDNN and RMSSD. HRV (frequency domain) was reported as LF (0.04–0.15 Hz) power (%), HF (0.15–0.40 Hz) power (%), LF/HF ratio, and natural logarithm of high frequency (LnHF) power.

### Other measurements

Perceived exertion with singing was reported by study subjects using the Borg Rating of Perceived Exertion (RPE) scale (46). The Borg RPE is a well-validated, quantitative scale used to assess an individual's level of exertion and is easy to understand. The scale ranges from 6 to 20, whereas 6 means “no exertion at all” and 20 means “maximal exertion”. At the end of each intervention, subjects were asked to rate their perceived level of exertion using this scale. The Borg RPE is the preferred method to assess intensity among those individuals who take medications that affect heart rate or pulse due to the scale's ability to capture exertion from central cardiovascular, respiratory, and nervous system functions (47). At the end of the singing and control visits, subjects were asked to rate their perceived exertion level using the Borg RPE.

### Sample size calculations

The pre-specified primary outcomes include three measures of vascular function. Brachial artery FMD% is the measure of macrovascular endothelial function. In a scenario with no anticipated differences, FMD correlations were observed to be 0.8 at 12 weeks (95% CI: 0.72, 0.87). Therefore, assuming an auto-regressive structure, the correlation would be 0.64 at 26 weeks and 0.41 at 52 weeks. Power analyses are based on a detectable FMD effect size that is clinically meaningful (48). For a 25% relative difference (2% absolute difference) in FMD with 90% power and alpha 0.05, we would need just 30 subjects (comparing singing interventions to control). But, to compare the singing interventions to each other, using a delta of 0.015, we need 52 subjects for 90% power and alpha 0.05. To have 52 evaluable subjects (assuming a 25% dropout rate), we enrolled 65 subjects. RHI and fRHI are measures of microvascular function. With a change of 0.08 with 0.1 standard deviation in RHI, a sample size of 52 would yield a power of 81% with alpha 0.05. Therefore, in a crossover design, we would be able to detect a change of 0.08 with power greater than 81% or we would be able to detect changes less than 0.08 with power equal to 81%. Similar remarks can be made for the fRHI.

### Statistical analysis

The Statistical Analysis System (SAS® 9.4) was used for all statistical analyses. Statistical methodology included outlier detection and removal with estimation of standard models for a three-treatment, crossover design. Because we have three primary outcome measures of vascular function, the significance level was adjusted to account for multiple comparisons using the Bonferroni correction, to minimize risk for type 1 error. The *p*-value for achieving statistical significance of our primary outcome was set at *p* < 0.016. Outcome results for RHI, fRHI, brachial artery FMD, and (secondary outcome) HRV are displayed as summary statistics by type of intervention: control, video instruction, and live music (by a music therapist). Outliers exceeding 2.5 standard deviations from the mean were removed to avoid unduly influential results; generally, the number of outliers removed were few for each outcome. General linear models for a crossover design with three treatments (49) were fit for both absolute and relative differences of each outcome. Primary analysis models estimated effects for the subject, visit number, treatment, and carry-over, if any. Analysis results are automatically adjusted for any significant carry-over effect of the interventions. Due to the COVID-19 pandemic, 20 out of 64 live music intervention visits were conducted by the music therapist remotely. Therefore, secondary analysis models included the primary effects along with virtual live music while the subject effect was partitioned by sex, race and age. Exploratory analyses included the secondary effects along with the video treatment partitioned by the four possible song choices (*Hey Jude, Jolene, This Land is Your Land, Amazing Grace).* These models were compared by rank-normalization (to assess the normality of the errors) and, similar results were found, i.e., the errors largely appeared to be approximately normal.

## Results

### Demographics

**Figure 1:**
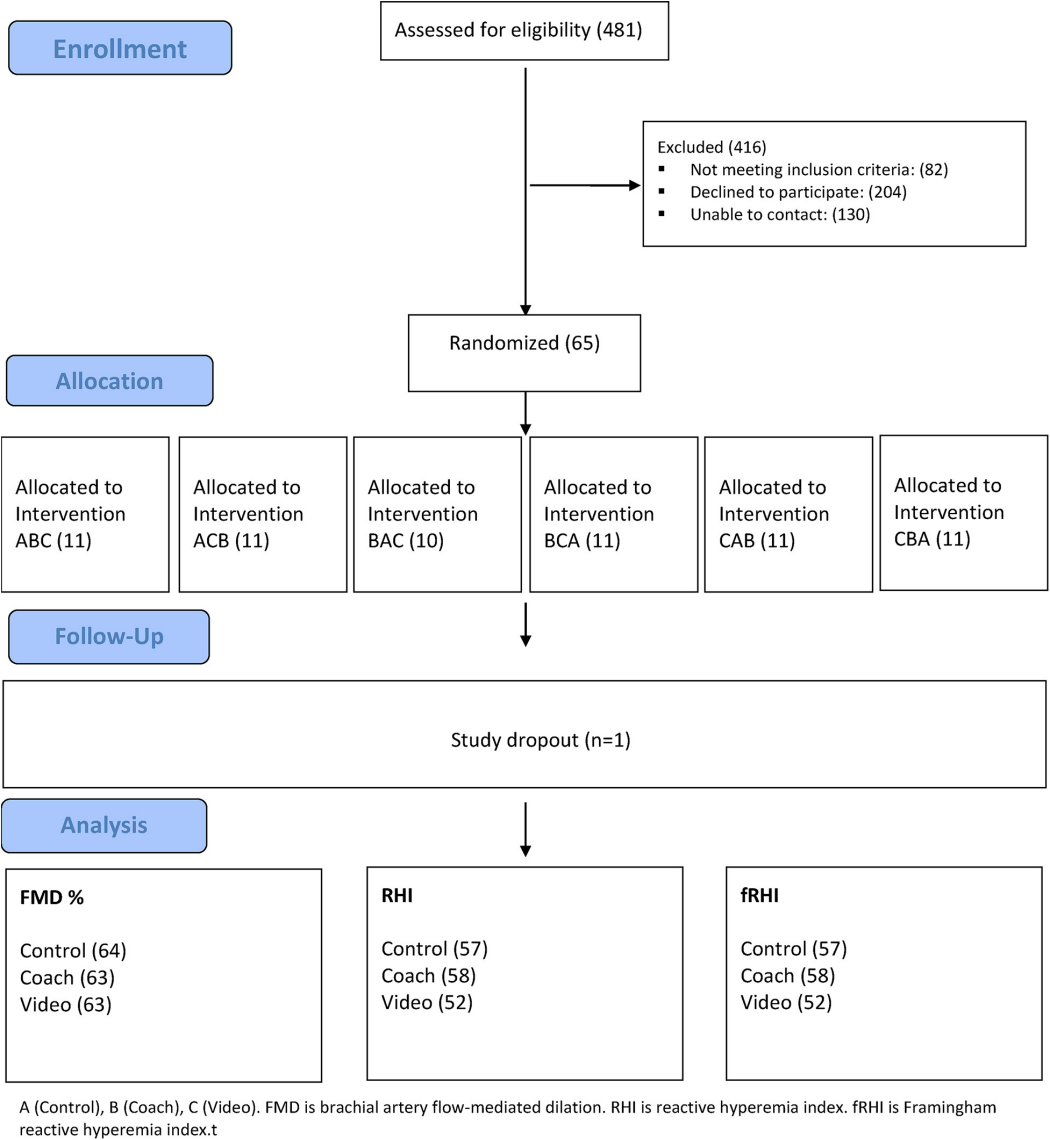
Consort diagram for randomized, cross-over, control trial.

Sixty-five subjects were enrolled between January 07, 2020 and August 18, 2023. A total of 481 patients were assessed for eligibility, Figure 1. Eighty-two patients were excluded because they either did not meet the inclusion criteria or had at least one exclusion criterion for the study. Another 334 patients either declined to participate (*n* = 204) or could not be reached after at least one attempt (*n* = 130). One participant withdrew after the first visit and, therefore, was not included in the final analysis. Table 1, stratified by gender, summarizes the overall demographic characteristics of the 65 subjects enrolled. The mean age of participants was 67.7 (±0.8) years with 40% female. Most of the participants were non-Hispanic (98.5%) and identified as White (86.2%). The mean body mass index (BMI) of the participants was 30.0 (±1.0) with 49.0% categorized as obese and an additional 26.2% as overweight. Physical limitation was ascertained by self-report with 53.8% reporting some level of orthopedic limitation.

**Table 1:** Baseline demographics and characteristics (*n =* 65).

Characteristic	Total *n* = 65 (col %)	Females *n* = 26 (col %)	Males *n* = 39 (col %)
Age, mean ± SD, years	67.7 ± 0.8	68.3 ± 1.4	67.3 ± 1.0
Non-Hispanic ethnicity	64 (98.5)	26 (100.0)	38 (100.0)
Race			
Black	7 (10.8)	3 (11.5)	4 (10.3)
White	56 (86.2)	23 (88.5)	33 (84.6)
Asian	1 (1.5)	0 (0.0)	1 (2.6)
Unknown	1 (1.5)	0 (0.0)	1 (2.6)
History of coronary artery disease			
Myocardial Infarction	41 (63.1)	11 (42.3)	30 (76.9)
Stent Placed	47 (74.6)	17 (70.8)	30 (76.9)
Coronary Artery Bypass	18 (27.7)	5 (19.2)	13 (33.3)
Diabetes Mellitus	19 (29.2)	9 (34.6)	10 (25.6)
Hypertension	49 (75.4)	20 (76.9)	29 (74.4)
High Cholesterol	55 (84.6)	21 (80.8)	34 (87.2)
Chronic Kidney Disease	10 (15.4)	4 (15.4)	6 (15.4)
Chronic Respiratory Disease	18 (27.7)	10 (38.5)	8 (20.5)
Heart Failure	12 (18.5)	8 (30.8)	4 (10.3)
Prior Smoking	26 (40.0)	12 (46.2)	14 (35.9)
BMI, mean ± SE	30.0 ± 1.0	30.5 ± 2.0	29.7 ± 1.1
BMI Category			
Underweight <18.5	4 (6.2)	3 (11.5)	1 (2.6)
Healthy weight 18.5–24.9	12 (18.5)	5 (19.2)	7 (17.9)
Overweight 25–29.9	17 (26.2)	6 (23.1)	11 (28.2)
Obese 30 or greater	32 (49.2)	12 (46.2)	20 (51.3)
Physical or orthopedic limitations	35 (53.8)	15 (57.7)	20 (51.3)
Level of limitation (*N* = 35)			
Minimal	21 (60.0)	7 (46.7)	14 (70.0)
Somewhat	12 (34.3)	8 (53.3)	4 (20.0)
Very	2 (5.7)	0 (0.0)	2 (10.0)
Ambulation aids (*N* = 35)	7 (20.0)	5 (33.3)	2 (10.0)

All participants had CAD, one of the mandatory inclusion criteria. History of MI was present in 63.1% of participants, coronary stenting in 74.6% and CABG in 27.7%. Amongst the comorbidities, dyslipidemia was most common and present in 84.6% of patients, followed by hypertension in 75.4%, and diabetes mellitus in another 29.2%. Forty percent of subjects were prior tobacco smokers with a mean cigarette pack year history of 15.5 (SE ± 2.6). All participants were non-smokers for at least one year prior to study enrollment.

Most characteristics including age, race, and comorbidities were balanced across both genders except for a few differences. More males had MI (76.9%) and history of CABG (33.3%) compared to females (42.3% and 19.2% respectively). Diabetes was more common among females compared to males (34.6% vs. 25.6%), as was heart failure (30.8% vs. 10.3%). Included females had a more extensive tobacco use history (mean pack years of 19.8 ± 4.9) compared to males (11.8 ± 2.2).

### Vascular function

The average pre intervention FMD% of the study participants was 4.2% (SE 0.19) highlighting the impaired baseline macrovascular endothelial function of the study population. The average baseline RHI and fRHI were 2.34 ± 0.05 and 2.20 ± 0.10, respectively. Unbalanced Analysis of Covariance (regression) was used to calculate the crossover estimates between the two different singing interventions and control as absolute change in post and pre-intervention FMD%, Table 2. For absolute change in FMD%, the live music intervention showed an estimated difference of −0.05 (SE: 0.42, *p* = 0.913) and the video intervention showed an estimated difference of −0.07 (SE: 0.42, *p* = 0.864) compared to the control intervention.

**Table 2:** Vascular function outcomes for singing interventions compared to control.

Parameter (Unit)	Absolute (Post-Pre)	
	Estimate (SE)	*P*-value
fRHI		
Live	0.11 (0.18)	0.570
Video	0.54 (0.19)	**0.005**
RHI		
Live	0.09 (0.12)	0.462
Video	0.13 (0.13)	0.290
FMD [%]		
Live	−0.05 (0.42)	0.913
Video	−0.07 (0.42)	0.864

Adjusted for the order of the intervention, carry over (if any), and period.

The bold value shows statistical significance.

**Figure 2:**
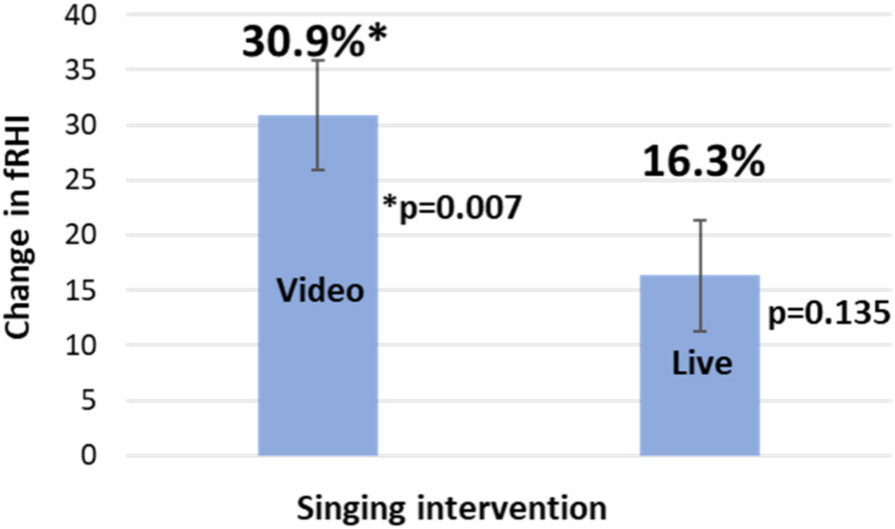
fRHI relative change (%) v. control (*n* = 65).

Twenty-five (out of 192 or 13%) PAT measurements were not included in the final analysis due to equipment malfunction, excessive signal artifact, leaking tubing, or incomplete occlusion. Microvascular endothelial function, as assessed by RHI and fRHI, showed a statistically significant improvement for the video intervention only. Compared to control, the video intervention showed an estimated increase of 0.54 (SE: 0.19, *p* = 0.005) for absolute change in fRHI (30.9 ± 11.1%, *p* = 0.007, relative change; Figure 2). However, the estimated difference between the live music intervention and control was not significant with an estimated increase of 0.11 (SE: 0.18, *p* = 0.570) for absolute change in fRHI. There was a statistically significant carry over effect observed with the video intervention (Estimate: 0.69, SE: 0.25, *p* = 0.007) but not with the live music intervention (Estimate: 0.17, SE: 0.25, *p* = 0.503). There was no significant change in RHI with either singing intervention, Table 2. For absolute change in RHI, the estimated difference was 0.09 (SE: 0.12, *p* = 0.462) for live music and 0.13 (SE: 0.13, *p* = 0.290) for the video intervention. Results of secondary and exploratory analysis models and carryover effects can be found in Supplementary Tables 2–4.

### Heart rate variability

HRV was assessed prior to, during, and after the intervention, with both during and post intervention values compared to pre intervention. Poor quality HRV recordings (5.7% pre-, 27.1% while singing, 21.2% post-singing) were excluded from the final analysis. The results for HRV are summarized in Tables 3, 4. There were no significant changes in the estimated differences for the time domain HRV parameters, SDNN and RMSSD, for either singing intervention compared to control in either post- to pre- or during to pre- intervention comparisons.

**Table 3:** Heart rate variability (post-Pre) outcomes for singing interventions compared to control.

Parameter (Unit)	Absolute (Post-Pre)		Relative (100*(Post-Pre)/Pre)	
	Estimate (SE)	*P*-value	Estimate (SE)	*P*-value
SDNN (ms)				
Live	3.33 (5.90)	0.575	−11.58 (22.32)	0.605
Video	2.64 (5.94)	0.658	−14.65 (22.47)	0.516
RMSSD (ms)				
Live	−6.73 (5.51)	0.225	−9.90 (57.04)	0.863
Video	−2.92 (5.55)	0.600	2.60 (57.45)	0.964
HF Power (%)				
Live	−6.88 (4.42)	0.124	−24.93 (23.80)	0.298
Video	−3.82 (4.55)	0.404	−44.80 (23.96)	0.065
LF Power (%)				
Live	5.99 (4.44)	0.181	6.02 (9.60)	0.532
Video	2.62 (4.57)	0.569	−0.61 (9.66)	0.950
LF/HF Ratio				
Live	1.34 (0.83)	0.109	43.05 (44.74)	0.339
Video	1.85 (0.84)	**0.030**	14.27 (45.42)	0.754
LnHF Power (ms^2^)				
Live	−0.62 (0.29)	**0.036**	−11.27 (6.00)	0.064
Video	−0.48 (0.30)	0.115	−10.42 (6.24)	0.099

Adjusted for the order of the intervention, carry over (if any), and period.

The bold value shows statistical significance.

**Table 4:** Heart rate variability (during-Pre) outcomes for singing interventions compared to control.

Parameter (Unit)	Absolute (During-Pre)		Relative (100*(During-Pre)/Pre)	
	Estimate (SE)	*P*-value	Estimate (SE)	*P*-value
SDNN (ms)				
Live	−7.39 (5.93)	0.217	−32.17 (18.10)	0.079
Video	−2.56 (6.15)	0.678	−24.53 (18.78)	0.195
RMSSD (ms)				
Live	−7.72 (6.05)	0.206	14.23 (50.31)	0.778
Video	−5.17 (6.16)	0.403	−14.47 (51.18)	0.778
HF Power (%)				
Live	3.04 (3.81)	0.427	10.48 (15.24)	0.494
Video	−5.59 (3.81)	0.147	−17.61 (15.25)	0.252
LF Power (%)				
Live	−4.21 (3.80)	0.272	−11.79 (8.95)	0.192
Video	4.53 (3.87)	0.246	1.94 (9.11)	0.832
LF/HF Ratio				
Live	0.43 (1.00)	0.670	1.96 (43.27)	0.964
Video	2.80 (1.03)	**0.008**	96.95 (44.53)	**0.033**
LnHF Power (ms^2^)				
Live	−0.55 (0.28)	0.050	−10.57 (5.52)	0.060
Video	−0.90 (0.29)	**0.003**	−15.63 (5.75)	**0.008**

Adjusted for the order of the intervention, carry over (if any), and period.

The bold value shows statistical significance.

For the frequency domain HRV parameters, the LF/HF ratio increased by 2.80 (SE: 1.03, *p* = 0.008), representing a relative change of 96.95% (SE: 44.53%, *p* = 0.033), for the video intervention compared to control (during to pre- change). When comparing post- to pre-intervention, the change in the LF/HF ratio remained significant (Estimate: 1.85, SE: 0.84, *p* = 0.030). There was no significant change in LF/HF ratio with the live music intervention. There were no significant changes in HF power and LF power for either singing intervention.

The estimated difference in LnHF power attained statistical significance for the video intervention (absolute change −0.90 ms^2^, SE: 0.29, *p* = 0.003; relative change: −15.63%, SE: 5.75, *p* = 0.008) but not for the live music intervention (absolute change: −0.55 ms^2^, SE: 0.28, *p* = 0.050; relative change: −10.57, SE: 5.52, *p* = 0.060) compared to control (during to pre-intervention comparison). However, when assessing post- to pre- change, it was the live music intervention that showed a statistically significant difference of −0.62 ms^2^ (SE: 0.29, *p* = 0.036) in LnHF power compared to control. Results of secondary and exploratory analysis models for all HRV values can be found in Supplementary Tables 5–16.

Compared to the video intervention, the live music intervention led to higher BORG RPE scores (9.98 ± 0.28 and 10.66 ± 0.29, respectively) with a difference of 0.68 (SE: 0.28), *p* = 0.0167. Self-reported level of enjoyment (scale 1–10 with 10 most enjoyable) was higher for the live music intervention compared to the video intervention (9.3 ± 0.13 and 8.5 ± 0.21; *p* = 0.0004).

## Discussion

In this randomized, researcher-blinded, crossover, control study of singing interventions in older adults with established CAD, 30 min of singing along to a pre-recorded instructional video improved microvascular, but not macrovascular, endothelial function. To our knowledge, this is the first rigorously conducted trial of singing interventions to demonstrate acute improvements in microvascular endothelial function in a population with baseline endothelial dysfunction (mean FMD 4.2 ± 0.33%). Findings advance the field of music medicine by providing insight into the mechanisms by which singing may improve health.

A hallmark of vascular aging is dysfunction of endothelial cells—the single layer of cells that lines all blood vessels and the heart and regulates exchanges between the blood and tissues. Healthy endothelial cells release nitric oxide which has vasodilatory and anti-thrombotic actions blocking the process of atherosclerosis. Endothelial dysfunction is considered an early precursor and common pathological feature of vascular diseases (50). For these reasons, direct measurement of vascular endothelial function is a powerful tool in translational science. These assessments have increasingly been applied in physiological studies to examine the mechanisms that underlie the acute or chronic impact of exposures that alter vascular function and risk for atherosclerosis (e.g., exercise training, smoking, hypercholesterolemia, hypertension, diet, medications). Vascular function strongly predicts cardiovascular events in patients with or without CVD (24), and endothelial dysfunction can be reversed by interventions known to reduce CVD risk (25, 26). For example, there is an 8–13% lower risk of CVD events per 1% increase in brachial artery FMD. For microvascular function, the RHI is provided by PAT. Meta-analyses show a 21% reduction in CVD events for every 0.1 natural log RHI (lnRHI) increase (51).

The microcirculation, constituted by pre-arterioles, arterioles, capillaries, and venules, is responsible for most of the resistance to flow that modulates blood pressure and tissue perfusion and has been increasingly recognized as a key feature of CVD (compared to the larger, “macro” vasculature) (52). Bonetti et al. measured digital pulse volume changes during reactive hyperemia in patients without obstructive CAD and either normal or abnormal coronary microvascular endothelial function (53). Multivariable analysis identified coronary blood flow responses to acetylcholine as the only independent predictor of RHI (53). Therefore, despite being measured at the periphery, the RHI could be used as a potential non-invasive tool to identify patients with coronary microvascular endothelial dysfunction. Based on a systematic review and meta-analysis evaluating the prognostic magnitude of non-invasive vascular function testing, the estimated increase in fRHI in our singing video intervention arm translates into an impressive 25% reduction in CVD risk (51). While RHI and fRHI are often highly correlated, their relationship to CVD risk factors may differ in important ways, and researchers are encouraged to present both (42, 54). While fRHI results are certainly promising, the next important step is to determine if this improvement in vascular function is sustained with a longer intervention period (weeks to months of singing). The sustained benefits of exercise are due to regular, intermittent bouts over time (repeated exposure to elevated blood flow), and we postulate that singing may be similar.

There was a lack of improvement in large-vessel brachial artery FMD. It may be that the singing stimulus was not large (or intense) enough to effect change. These mean Borg RPE scores in both singing interventions equate to light-intensity physical activity (less than 3 metabolic equivalents or METs or 25–44% VO2max) (46). A meta-analysis concluded that higher intensity physical activities are likely needed to see a meaningful change in FMD in middle-aged and elderly people (55). In addition, more habitual activity over weeks to months, is more important from a prognosis standpoint and evaluation of sustained vascular adaptation should be tested in future clinical trials of singing. It is possible that the timing of the FMD measurement after singing (or exercise) matters. For example, studies taking multiple FMD measurements after a bout of moderate or vigorous-intensity exercise show an immediate decrease in FMD followed by a normal or supranormal FMD response and ultimate normalization within 24–48 h after exercise (35). Higher exercise intensities (>80% VO2max) resulted in a larger decrease in FMD post-exercise whereas most, but not all, studies of low-moderate intensity exercise reported an increase in FMD after exercise. We measured FMD approximately 40 min after singing. Based on extrapolation from studies of exercise, this would fall within the rise (of the initial drop) in FMD. This timing may have influenced results, and future studies of singing should explore this potential biphasic FMD response.

The participants in the live music intervention with the music therapist reported a higher level of enjoyment which may be due to their ability to adapt their singing more effectively to align with the therapist's advice. The lack of improvement in vascular function, therefore, with the live music visit with a music therapist (compared to control) was unexpected. It may be that a single visit singing with someone you just met could be anxiety-provoking, especially for amateur singers. A longer-term singing intervention or program could likely ameliorate this issue. Due to the COVID-19 pandemic, 20 out of 64 (31%) live music intervention visits were conducted by the music therapist remotely. Therefore, secondary analysis models included the primary effects along with unanticipated virtual live music. The virtual live music sessions had a lower effect size (5.2%) compared to the in-person live music sessions (14.2%) though we are underpowered to separate these for significance (Supplementary Table 1). Music structure (e.g., tempo, rhythm, dynamics) is complex, and it is possible that these elements could differentially impact physiological signals. With 40 song choices for the live music visit, it is impossible to evaluate this. However, the video visit included just 4 song choices (and subjects selected 2). In a strictly exploratory analysis, we found that *Amazing Grace* had the largest effect size at 22.3% and *This Land is Your Land* had the lowest at 10.1% (Supplementary Table 1). These were not statistically significant findings but might be hypothesis-generating for future research in music medicine.

There was a carryover effect with the instructional video visit that is included in the analysis of crossover studies when detected (Supplementary Table 2). This likely means that a minimum of 2 days for potential washout from the previous visit was not long enough. One possible explanation for this carryover effect is the “earworm”—the experience of a song that repeats persistently in the mind—is a ubiquitous yet mysterious cognitive phenomenon, manifested as “inner singing” and may result in continued singing after the intervention (56). We did not ask subjects if they continued to sing in the days following their interventions, but this may be important to consider in future studies. Interestingly, functional magnetic resonance imaging (fMRI) imaging of the brain in soprano Reneé Fleming in 2017 demonstrated that thinking about singing activated the brain to a larger extent than did singing itself. This provides some proof of concept on the potential for earworms to have a physiological effect; however, more definitive studies are needed.

The acute HRV changes observed with the video intervention in our study, specifically increases in the LF/HF ratio and reductions in the LnHF power, are similar to those of light-intensity exercise activity whereby light exercise elicits a slight increase in sympathetic nervous system activity (57, 58). In general, an increase in LF power implies a more dominant activity of the sympathetic nervous system (SNS) while an increased power in the high frequency band indicates stronger influence of the parasympathetic nervous system (PNS). Pagani et al. proposed the LF/HF ratio as an index of sympathovagal balance between the two nervous systems (59). Vagal activity is the major contributor to the HF component, and LnHF power is posited to reflect vagal tone (60), that decreased during our singing interventions. If we extrapolate what we know about regular exercise, it is likely that a longer intervention of singing (weeks to months) would lead to improved resting HRV due to enhanced autonomic nervous system balance (61). This may be especially important in an older population, as HRV is known to worsen with aging. These chronic alterations in HRV are likely more clinically meaningful than acute changes and should be incorporated into future studies of singing.

The strengths of the present study include the randomized crossover with control intervention trial design. The crossover design has the advantage of inherently controlling for subject-level variables (each subject serves as their own control) thereby reducing risk of confounding dramatically. Furthermore, it did expose carry-over effects with the instructional singing video intervention. Because it is difficult to know what an appropriate washout period is for a singing intervention, future studies could consider alternative clinical trial designs and/or longer (than 7 days) of washout. Regarding HRV measurements, the accuracy of these measurements (with the equipment we used) during active singing, compared to rest, is questionable, possibly due to movement or perspiration (62). Therefore, commercial equipment used to assess HRV should satisfy industry standards in terms of signal-to-noise ratio and other performance metrics (60). It is also important to note that our study included a music therapist in the live singing intervention and a voice professor in the pre-recorded video intervention, which represent two distinct professions. We compared each intervention independently to the control (rest) group, as our study was not powered to compare these two interventions directly. Future studies should explore differences between live and recorded singing directed by the same music professional to minimize confounding by profession (music therapist or voice professor). Breathing has been consistently shown to be a major actor in determining physiological changes associated with singing and may be influenced by song structure (22). Therefore, respiratory rate during singing should be accounted for in future singing studies, especially those studies measuring HRV. Finally, the study population may not adequately represent the broader demographic of older adults with CAD in terms of race and ethnicity; however, 44% of the study population were females.

## Conclusions

Singing along to a pre-recorded instructional video for 30 min improved microvascular, but not macrovascular, endothelial function, in older patients with known CAD. Singing should be considered as an accessible and safe therapeutic intervention in an older population who otherwise may have physical or orthopedic limitations hindering participation in traditional exercise. Future studies need to explore the sustained vascular response to singing over weeks to months and explore the potential for “earworm” effects between visits.

## Data Availability

The raw data supporting the conclusions of this article will be made available by the authors, without undue reservation.
