# Supplementary material for: Effects of singing on vascular health in older adults with coronary artery disease: a randomized, crossover trial
Source: Front Cardiovasc Med. 2025 Apr 28;12:1546462. doi: 10.3389/fcvm.2025.1546462 (PMC12066452; doi:10.3389/fcvm.2025.1546462)
Supplement: Supplementary file 1 [file Table1.docx]

| **Supplementary Table 1. List of songs for live music visit.** | | | | | | |
| --- | --- | --- | --- | --- | --- | --- |
|  | **No** | **Song Name** |  | **No** | **Song Name** |  |
|  | 1 | Don’t Sit Under the Apple Tree (Andrew Sisters) |  | 21 | Don’t Stop Believin’ (Journey) |  |
|  | 2 | Five Foot Two, Eyes of Blue (Dean Martin) |  | 22 | Piano Man (Billy Joel) |  |
|  | 3 | Side by Side (Harry Woods) |  | 23 | Billy Jean (Michael Jackson) |  |
|  | 4 | When the Red, Red Robin Comes Bob, Bob, Bobbin Along (Al Jolson) |  | 24 | Free Fallin’ (Tom Petty and the Heartbreakers) |  |
|  | 5 | Let me Call You Sweetheart (Bing Crosby) |  | 25 | Livin’ on a Prayer (Bon Jovi) |  |
|  | 6 | All I Have to Do is Dream (Everly Brothers) |  | 26 | Don’t Fence Me in (Roy Rogers) |  |
|  | 7 | Fly Me to the Moon (Frank Sinatra) |  | 27 | Crazy (Patsy Cline) |  |
|  | 8 | Hound Dog (Elvis Presley) |  | 28 | Hey Good Lookin’ (Hank Williams Snr.) |  |
|  | 9 | Rockin’ Robin (Bobby Day) |  | 29 | Leavin’ on a Jet Plane (John Denver) |  |
|  | 10 | Rock Around the Clock (Bill Hayley) |  | 30 | Ring of Fire (Johnny Cash) |  |
|  | 11 | Happy Together (The Turtles) |  | 31 | How Great Thou Art |  |
|  | 12 | My Girl (The Temptations) |  | 32 | Swing Low, Sweet Chariot |  |
|  | 13 | Stand by Me (Ben E. King) |  | 33 | He’s Got the Whole World in His Hands |  |
|  | 14 | I Want to Hold Your Hand (The Beatles) |  | 34 | God Bless America |  |
|  | 15 | Under the Boardwalk (The Drifters) |  | 35 | Home on the Range |  |
|  | 16 | Hotel California (Eagles) |  | 36 | This Land is Your Land |  |
|  | 17 | Lean on Me (Bill Withers) |  | 37 | You Are My Sunshine |  |
|  | 18 | I Can See Clearly Now (Johnny Nash) |  | 38 | Take Me Out to the Ballgame |  |
|  | 19 | Cecilia (Simon & Garfunkel) |  | 39 | The Happy Wanderer |  |
|  | 20 | Crocodile Rock (Elton John |  | 40 | Beer Barrel Polka |  |

| **Supplementary Table 2. Vascular function secondary and exploratory analyses - fRHI compared to control** | | | | | | | | | |
| --- | --- | --- | --- | --- | --- | --- | --- | --- | --- |
|  |  | **Absolute** (post-pre) | | |  | **Relative** (100*(post-pre)/pre) | | |  |
| **Model/ Parameter** |  | **Estimate** | **Std-Err** | **P-value** |  | **Estimate** | **Std-Err** | **P-value** |  |
| **fRHI - Secondary Analysis** |  |  |  |  |  |  |  |  |  |
| **Virtual Live** |  | -0.11 | 0.30 | 0.712 |  | 5.24 | 17.58 | 0.766 |  |
| **In-Person Live** |  | 0.15 | 0.22 | 0.500 |  | 14.21 | 12.89 | 0.273 |  |
| **Video** |  | 0.55 | 0.19 | 0.005 |  | 30.77 | 11.17 | 0.007 |  |
| **Carry In-Person Live** |  | 0.17 | 0.25 | 0.503 |  | 14.23 | 14.85 | 0.341 |  |
| **Carry Video** |  | 0.69 | 0.25 | 0.008 |  | 34.49 | 14.71 | 0.021 |  |
| **fRHI - Exploratory Analysis** |  |  |  |  |  |  |  |  |  |
| **Song** |  |  |  |  |  |  |  |  |  |
| Hey Jude |  | 0.26 | 0.25 | 0.288 |  | 14.38 | 14.48 | 0.323 |  |
| Jolene |  | 0.39 | 0.37 | 0.300 |  | 16.90 | 21.71 | 0.439 |  |
| This Land is Your Land |  | 0.14 | 0.25 | 0.563 |  | 10.09 | 14.41 | 0.486 |  |
| Amazing Grace |  | 0.38 | 0.27 | 0.162 |  | 22.27 | 15.97 | 0.167 |  |
| **Virtual Live** |  | -0.11 | 0.30 | 0.713 |  | 5.29 | 17.86 | 0.768 |  |
| **In-Person Live** |  | 0.14 | 0.23 | 0.523 |  | 14.35 | 13.19 | 0.280 |  |
| **Carry In-Person Live** |  | 0.16 | 0.27 | 0.553 |  | 14.65 | 15.62 | 0.351 |  |
| **Carry Video** |  | 0.68 | 0.26 | 0.009 |  | 34.95 | 15.08 | 0.023 |  |
| Adjusted for the order of the intervention, carry over, and period. | | | | | | | | | |

| **Supplementary Table 3. Vascular function secondary and exploratory analyses - RHI compared to control** | | | | | | | | | |
| --- | --- | --- | --- | --- | --- | --- | --- | --- | --- |
|  |  | **Absolute** (post-pre) | | |  | **Relative** (100*(post-pre)/pre) | | |  |
| **Model/ Parameter** |  | **Estimate** | **Std-Err** | **P-value** |  | **Estimate** | **Std-Err** | **P-value** |  |
| **RHI - Secondary Analysis** |  |  |  |  |  |  |  |  |  |
| **Virtual Live** |  | -0.21 | 0.22 | 0.348 |  | -5.20 | 10.38 | 0.618 |  |
| **In-Person Live** |  | 0.17 | 0.15 | 0.255 |  | 8.89 | 7.00 | 0.208 |  |
| **Video** |  | 0.14 | 0.13 | 0.278 |  | 8.31 | 5.95 | 0.166 |  |
| **RHI - Exploratory Analysis** |  |  |  |  |  |  |  |  |  |
| **Song** |  |  |  |  |  |  |  |  |  |
| Hey Jude |  | 0.08 | 0.18 | 0.634 |  | 2.43 | 8.40 | 0.773 |  |
| Jolene |  | 0.09 | 0.27 | 0.742 |  | 8.49 | 12.77 | 0.508 |  |
| This Land is Your Land |  | -0.06 | 0.18 | 0.729 |  | -0.74 | 8.36 | 0.930 |  |
| Amazing Grace |  | 0.19 | 0.19 | 0.320 |  | 9.91 | 9.17 | 0.283 |  |
| **Virtual Live** |  | -0.20 | 0.22 | 0.359 |  | -5.25 | 10.52 | 0.619 |  |
| **In-Person Live** |  | 0.17 | 0.15 | 0.265 |  | 8.90 | 7.10 | 0.213 |  |
| Adjusted for the order of the intervention, carry over, and period. | | | | | | | | | |

| **Supplementary Table 4. Vascular function secondary and exploratory analyses – FMD% compared to control** | | | | | | | | | |
| --- | --- | --- | --- | --- | --- | --- | --- | --- | --- |
|  |  | **Absolute** (post-pre) | | |  | **Relative** (100*(post-pre)/pre) | | |  |
| **Model/ Parameter** |  | **Estimate** | **Std-Err** | **P-value** |  | **Estimate** | **Std-Err** | **P-value** |  |
| **FMD% - Secondary Analysis** |  |  |  |  |  |  |  |  |  |
| **Virtual Live** |  | 0.97 | 0.78 | 0.213 |  | -70.42 | 101.49 | 0.489 |  |
| **In-Person Live** |  | -0.37 | 0.49 | 0.457 |  | -11.22 | 64.70 | 0.863 |  |
| **Video** |  | -0.08 | 0.42 | 0.856 |  | -122.79 | 55.19 | 0.028 |  |
| **FMD% - Exploratory Analysis** |  |  |  |  |  |  |  |  |  |
| **Song** |  |  |  |  |  |  |  |  |  |
| Hey Jude |  | -0.91 | 0.61 | 0.139 |  | -149.30 | 78.46 | 0.060 |  |
| Jolene |  | 0.13 | 0.92 | 0.891 |  | -243.21 | 117.58 | 0.041 |  |
| This Land is Your Land |  | 0.03 | 0.61 | 0.957 |  | -14.86 | 78.64 | 0.850 |  |
| Amazing Grace |  | 0.55 | 0.65 | 0.403 |  | 21.88 | 83.34 | 0.793 |  |
| **Virtual Live** |  | 0.94 | 0.78 | 0.231 |  | -63.33 | 99.81 | 0.527 |  |
| **In-Person Live** |  | -0.37 | 0.50 | 0.452 |  | -16.68 | 63.51 | 0.793 |  |
| Adjusted for the order of the intervention, carry over, and period. | | | | | | | | | |

| **Supplementary Table 5. Heart rate variability secondary and exploratory analyses - SDNN compared to control** | | | | | | | | | |
| --- | --- | --- | --- | --- | --- | --- | --- | --- | --- |
|  |  | **Absolute** (post-pre) | | |  | **Relative** (100*(post-pre)/pre) | | |  |
| **Model/ Parameter** |  | **Estimate** | **Std-Err** | **P-value** |  | **Estimate** | **Std-Err** | **P-value** |  |
| **SDNN - Secondary Analysis** |  |  |  |  |  |  |  |  |  |
| **Virtual Live** |  | -6.83 | 10.29 | 0.509 |  | -7.91 | 39.02 | 0.840 |  |
| **In-Person Live** |  | 6.54 | 7.65 | 0.395 |  | -7.87 | 28.99 | 0.787 |  |
| **Video** |  | 2.60 | 5.96 | 0.663 |  | -14.69 | 22.61 | 0.518 |  |
| **SDNN – Exploratory Analysis** |  |  |  |  |  |  |  |  |  |
| **Song** |  |  |  |  |  |  |  |  |  |
| Hey Jude |  | 2.36 | 9.34 | 0.801 |  | -7.76 | 35.38 | 0.827 |  |
| Jolene |  | 3.96 | 13.99 | 0.778 |  | 12.25 | 53.02 | 0.818 |  |
| This Land is Your Land |  | -0.74 | 9.27 | 0.937 |  | -12.69 | 35.13 | 0.719 |  |
| Amazing Grace |  | 1.86 | 9.83 | 0.850 |  | -7.46 | 37.26 | 0.842 |  |
| **Virtual Live** |  | -6.53 | 10.60 | 0.540 |  | -7.80 | 40.16 | 0.846 |  |
| **In-Person Live** |  | 6.33 | 7.83 | 0.422 |  | -8.27 | 29.69 | 0.781 |  |
| Adjusted for the order of the intervention, carry over, and period. Units for SDNN are milliseconds. | | | | | | | | | |

| **Supplementary Table 6. Heart rate variability secondary and exploratory analyses - RMSSD compared to control** | | | | | | | | | |
| --- | --- | --- | --- | --- | --- | --- | --- | --- | --- |
|  |  | **Absolute** (post-pre) | | |  | **Relative** (100*(post-pre)/pre) | | |  |
| **Model/ Parameter** |  | **Estimate** | **Std-Err** | **P-value** |  | **Estimate** | **Std-Err** | **P-value** |  |
| **RMSSD - Secondary Analysis** |  |  |  |  |  |  |  |  |  |
| **Virtual Live** |  | 1.03 | 9.53 | 0.914 |  | 49.14 | 98.56 | 0.619 |  |
| **In-Person Live** |  | -7.22 | 7.17 | 0.317 |  | -33.32 | 74.10 | 0.654 |  |
| **Video** |  | -2.93 | 5.59 | 0.602 |  | 2.22 | 57.73 | 0.969 |  |
| **RMSSD - Exploratory Analysis** |  |  |  |  |  |  |  |  |  |
| **Song** |  |  |  |  |  |  |  |  |  |
| Hey Jude |  | -4.23 | 8.20 | 0.608 |  | 36.17 | 83.67 | 0.667 |  |
| Jolene |  | 7.24 | 12.56 | 0.566 |  | 48.25 | 128.2 | 0.708 |  |
| This Land is Your Land |  | -11.73 | 8.23 | 0.158 |  | -150.55 | 83.95 | 0.077 |  |
| Amazing Grace |  | 9.09 | 8.79 | 0.304 |  | 121.69 | 89.69 | 0.179 |  |
| **Virtual Live** |  | 2.04 | 9.70 | 0.834 |  | 73.60 | 99.03 | 0.460 |  |
| **In-Person Live** |  | -7.71 | 7.27 | 0.292 |  | -46.45 | 74.21 | 0.533 |  |
| Adjusted for the order of the intervention, carry over, and period. Units for RMSSD are in milliseconds. | | | | | | | | | |

| **Supplementary Table 7. Heart rate variability secondary and exploratory analyses - HF Power % compared to control** | | | | | | | | | |
| --- | --- | --- | --- | --- | --- | --- | --- | --- | --- |
|  |  | **Absolute** (post-pre) | | |  | **Relative** (100*(post-pre)/pre) | | |  |
| **Model/ Parameter** |  | **Estimate** | **Std-Err** | **P-value** |  | **Estimate** | **Std-Err** | **P-value** |  |
| **HF Power %**  **Secondary Analysis** |  |  |  |  |  |  |  |  |  |
| **Virtual Live** |  | 6.99 | 6.75 | 0.304 |  | 89.37 | 39.79 | 0.028 |  |
| **In-Person Live** |  | -10.24 | 5.48 | 0.066 |  | -67.06 | 29.84 | 0.027 |  |
| **Video** |  | -3.99 | 4.55 | 0.383 |  | -45.91 | 23.37 | 0.053 |  |
| **Carry In-Person Live** |  | -6.54 | 5.97 | 0.277 |  |  |  |  |  |
| **Carry Video** |  | 7.12 | 6.06 | 0.244 |  |  |  |  |  |
| **HF Power %**  **Exploratory Analysis** |  |  |  |  |  |  |  |  |  |
| **Song** |  |  |  |  |  |  |  |  |  |
| Hey Jude |  | 0.86 | 6.03 | 0.887 |  | -27.25 | 34.56 | 0.433 |  |
| Jolene |  | -8.44 | 9.05 | 0.354 |  | -19.68 | 52.69 | 0.710 |  |
| This Land is Your Land |  | -4.52 | 6.01 | 0.455 |  | -63.48 | 34.64 | 0.071 |  |
| Amazing Grace |  | 0.97 | 6.49 | 0.881 |  | 24.48 | 36.99 | 0.510 |  |
| **Virtual Live** |  | 7.74 | 6.92 | 0.267 |  | 94.33 | 40.51 | 0.023 |  |
| **In-Person Live** |  | -10.07 | 5.59 | 0.076 |  | -68.42 | 30.22 | 0.027 |  |
| **Carry In-Person Live** |  | -5.40 | 6.13 | 0.382 |  |  |  |  |  |
| **Carry Video** |  | 7.48 | 6.15 | 0.228 |  |  |  |  |  |
| Adjusted for the order of the intervention, carry over, and period. | | | | | | | | | |

| **Supplementary Table 8. Heart rate variability secondary and exploratory analyses - LF Power % compared to control** | | | | | | | | | |
| --- | --- | --- | --- | --- | --- | --- | --- | --- | --- |
|  |  | **Absolute** (post-pre) | | |  | **Relative** (100*(post-pre)/pre) | | |  |
| **Model/ Parameter** |  | **Estimate** | **Std-Err** | **P-value** |  | **Estimate** | **Std-Err** | **P-value** |  |
| **LF Power %**  **Secondary Analysis** |  |  |  |  |  |  |  |  |  |
| **Virtual Live** |  | -6.74 | 6.79 | 0.324 |  | -8.57 | 16.53 | 0.606 |  |
| **In-Person Live** |  | 9.23 | 5.51 | 0.098 |  | 10.06 | 12.40 | 0.420 |  |
| **Video** |  | 2.78 | 4.58 | 0.545 |  | -0.50 | 9.71 | 0.959 |  |
| **Carry In-Person Live** |  | 6.01 | 6.00 | 0.320 |  |  |  |  |  |
| **Carry Video** |  | -6.68 | 6.09 | 0.276 |  |  |  |  |  |
| **LF Power %**  **Exploratory Analysis** |  |  |  |  |  |  |  |  |  |
| **Song** |  |  |  |  |  |  |  |  |  |
| Hey Jude |  | -0.08 | 5.93 | 0.990 |  | 2.16 | 14.13 | 0.879 |  |
| Jolene |  | 11.28 | 9.17 | 0.222 |  | 13.07 | 21.83 | 0.551 |  |
| This Land is Your Land |  | 3.92 | 5.93 | 0.511 |  | 4.04 | 14.12 | 0.776 |  |
| Amazing Grace |  | -1.48 | 6.43 | 0.819 |  | -11.59 | 15.31 | 0.451 |  |
| **Virtual Live** |  | -7.32 | 7.11 | 0.306 |  | -8.96 | 16.92 | 0.598 |  |
| **In-Person Live** |  | 6.69 | 5.31 | 0.212 |  | 9.46 | 12.64 | 0.457 |  |
| Adjusted for the order of the intervention, carry over, and period. | | | | | | | | | |

| **Supplementary Table 9. Heart rate variability secondary and exploratory analyses – LF/HF Ratio compared to control** | | | | | | | | | |
| --- | --- | --- | --- | --- | --- | --- | --- | --- | --- |
|  |  | **Absolute** (post-pre) | | |  | **Relative** (100*(post-pre)/pre) | | |  |
| **Model/ Parameter** |  | **Estimate** | **Std-Err** | **P-value** |  | **Estimate** | **Std-Err** | **P-value** |  |
| **LF/HF Ratio**  **Secondary Analysis** |  |  |  |  |  |  |  |  |  |
| **Virtual Live** |  | -1.60 | 1.42 | 0.264 |  | 34.38 | 77.33 | 0.658 |  |
| **In-Person Live** |  | 2.09 | 1.06 | 0.053 |  | 26.82 | 57.92 | 0.645 |  |
| **Video** |  | 1.86 | 0.84 | 0.029 |  | 14.05 | 45.66 | 0.759 |  |
| **LF/HF Ratio**  **Exploratory Analysis** |  |  |  |  |  |  |  |  |  |
| **Song** |  |  |  |  |  |  |  |  |  |
| Hey Jude |  | 1.55 | 1.22 | 0.208 |  | 43.62 | 66.01 | 0.511 |  |
| Jolene |  | 1.03 | 1.88 | 0.586 |  | 124.64 | 101.38 | 0.223 |  |
| This Land is Your Land |  | 1.92 | 1.23 | 0.124 |  | -31.39 | 66.55 | 0.639 |  |
| Amazing Grace |  | -0.67 | 1.31 | 0.610 |  | -19.92 | 70.91 | 0.780 |  |
| **Virtual Live** |  | -1.67 | 1.45 | 0.253 |  | 41.50 | 78.14 | 0.597 |  |
| **In-Person Live** |  | 2.08 | 1.08 | 0.058 |  | 18.27 | 58.39 | 0.755 |  |
| Adjusted for the order of the intervention, carry over, and period. | | | | | | | | | |

| **Supplementary Table 10. Heart rate variability secondary and exploratory analyses - Ln HF Power compared to control** | | | | | | | | | |
| --- | --- | --- | --- | --- | --- | --- | --- | --- | --- |
|  |  | **Absolute** (post-pre) | | |  | **Relative** (100*(post-pre)/pre) | | |  |
| **Model/ Parameter** |  | **Estimate** | **Std-Err** | **P-value** |  | **Estimate** | **Std-Err** | **P-value** |  |
| **Ln HF Power**  **Secondary Analysis** |  |  |  |  |  |  |  |  |  |
| **Virtual Live** |  | -0.08 | 0.45 | 0.851 |  | -0.73 | 9.24 | 0.937 |  |
| **In-Person Live** |  | -0.58 | 0.36 | 0.113 |  | -10.92 | 7.46 | 0.147 |  |
| **Video** |  | -0.48 | 0.30 | 0.119 |  | -10.41 | 6.28 | 0.101 |  |
| **Carry In-Person Live** |  | -0.97 | 0.41 | 0.020 |  | -17.81 | 8.36 | 0.037 |  |
| **Carry Video** |  | -0.85 | 0.41 | 0.040 |  | -16.35 | 8.35 | 0.054 |  |
| **Ln HF Power**  **Exploratory Analysis** |  |  |  |  |  |  |  |  |  |
| **Song** |  |  |  |  |  |  |  |  |  |
| Hey Jude |  | -0.12 | 0.39 | 0.752 |  | -5.17 | 8.06 | 0.524 |  |
| Jolene |  | 0.04 | 0.60 | 0.953 |  | 5.28 | 12.23 | 0.667 |  |
| This Land is Your Land |  | -0.76 | 0.39 | 0.058 |  | -17.24 | 8.09 | 0.037 |  |
| Amazing Grace |  | 0.15 | 0.43 | 0.733 |  | 4.44 | 8.73 | 0.612 |  |
| **Virtual Live** |  | -0.00 | 0.46 | 0.993 |  | 0.75 | 9.36 | 0.936 |  |
| **In-Person Live** |  | -0.62 | 0.37 | 0.097 |  | -11.71 | 7.55 | 0.125 |  |
| **Video** |  | 0.0 |  |  |  | 0.0 |  |  |  |
| **Carry In-Person Live** |  | -0.94 | 0.42 | 0.026 |  | -17.56 | 8.52 | 0.043 |  |
| **Carry Video** |  | -0.84 | 0.41 | 0.044 |  | -16.32 | 8.39 | 0.056 |  |
| Adjusted for the order of the intervention, carry over, and period. Units for Ln HF Power are milliseconds squared (ms^2^). | | | | | | | | | |

| **Supplementary Table 11. Heart rate variability secondary and exploratory analyses - SDNN compared to control** | | | | | | | | | |
| --- | --- | --- | --- | --- | --- | --- | --- | --- | --- |
|  |  | **Absolute** (during-pre) | | |  | **Relative** (100*(during-pre)/pre) | | |  |
| **Model/ Parameter** |  | **Estimate** | **Std-Err** | **P-value** |  | **Estimate** | **Std-Err** | **P-value** |  |
| **SDNN - Secondary Analysis** |  |  |  |  |  |  |  |  |  |
| **Virtual Live** |  | -9.81 | 10.29 | 0.344 |  | -8.61 | 31.59 | 0.786 |  |
| **In-Person Live** |  | -2.89 | 7.58 | 0.704 |  | -28.23 | 23.26 | 0.229 |  |
| **Video** |  | -2.52 | 6.16 | 0.683 |  | -24.49 | 18.90 | 0.199 |  |
| **SDNN – Exploratory Analysis** |  |  |  |  |  |  |  |  |  |
| **Song** |  |  |  |  |  |  |  |  |  |
| Hey Jude |  | -7.00 | 9.39 | 0.459 |  | -12.79 | 28.86 | 0.659 |  |
| Jolene |  | 3.03 | 13.62 | 0.825 |  | 7.62 | 41.88 | 0.856 |  |
| This Land is Your Land |  | -3.42 | 9.34 | 0.716 |  | -21.45 | 28.71 | 0.457 |  |
| Amazing Grace |  | 4.11 | 10.00 | 0.682 |  | -9.25 | 30.74 | 0.764 |  |
| **Virtual Live** |  | -10.37 | 10.58 | 0.330 |  | -8.53 | 32.52 | 0.794 |  |
| **In-Person Live** |  | -2.51 | 7.75 | 0.747 |  | -28.47 | 23.83 | 0.236 |  |
| Adjusted for the order of the intervention, carry over, and period. Units for SDNN are milliseconds. | | | | | | | | | |

| **Supplementary Table 12. Heart rate variability secondary and exploratory analyses - RMSSD compared to control** | | | | | | | | | |
| --- | --- | --- | --- | --- | --- | --- | --- | --- | --- |
|  |  | **Absolute** (during-pre) | | |  | **Relative** (100*(during-pre)/pre) | | |  |
| **Model/ Parameter** |  | **Estimate** | **Std-Err** | **P-value** |  | **Estimate** | **Std-Err** | **P-value** |  |
| **RMSSD - Secondary Analysis** |  |  |  |  |  |  |  |  |  |
| **Virtual Live** |  | -0.69 | 10.42 | 0.947 |  | 87.66 | 86.04 | 0.311 |  |
| **In-Person Live** |  | -7.40 | 7.76 | 0.343 |  | -26.17 | 64.05 | 0.684 |  |
| **Video** |  | -5.16 | 6.20 | 0.408 |  | -15.84 | 51.18 | 0.758 |  |
| **RMSSD - Exploratory Analysis** |  |  |  |  |  |  |  |  |  |
| **Song** |  |  |  |  |  |  |  |  |  |
| Hey Jude |  | -13.21 | 8.96 | 0.145 |  | 16.42 | 74.71 | 0.827 |  |
| Jolene |  | 6.22 | 13.42 | 0.644 |  | 28.27 | 111.88 | 0.801 |  |
| This Land is Your Land |  | -10.44 | 9.03 | 0.251 |  | -87.39 | 75.26 | 0.249 |  |
| Amazing Grace |  | 11.67 | 9.66 | 0.231 |  | 45.45 | 80.52 | 0.574 |  |
| **Virtual Live** |  | -0.99 | 10.55 | 0.925 |  | 99.77 | 87.93 | 0.260 |  |
| **In-Person Live** |  | -6.84 | 7.83 | 0.385 |  | -33.13 | 65.23 | 0.613 |  |
| Adjusted for the order of the intervention, carry over, and period. Units for RMSSD are in milliseconds. | | | | | | | | | |

| **Supplementary Table 13. Heart rate variability secondary and exploratory analyses - HF Power % compared to control** | | | | | | | | | |
| --- | --- | --- | --- | --- | --- | --- | --- | --- | --- |
|  |  | **Absolute** (during-pre) | | |  | **Relative** (100*(during-pre)/pre) | | |  |
| **Model/ Parameter** |  | **Estimate** | **Std-Err** | **P-value** |  | **Estimate** | **Std-Err** | **P-value** |  |
| **HF Power %**  **Secondary Analysis** |  |  |  |  |  |  |  |  |  |
| **Virtual Live** |  | 2.60 | 6.61 | 0.695 |  | 28.69 | 26.24 | 0.278 |  |
| **In-Person Live** |  | 1.77 | 5.01 | 0.725 |  | -3.52 | 19.89 | 0.860 |  |
| **Video** |  | -5.65 | 3.84 | 0.145 |  | -18.26 | 15.25 | 0.235 |  |
| **HF Power %**  **Exploratory Analysis** |  |  |  |  |  |  |  |  |  |
| **Song** |  |  |  |  |  |  |  |  |  |
| Hey Jude |  | -3.19 | 5.57 | 0.569 |  | -3.18 | 21.58 | 0.883 |  |
| Jolene |  | -0.15 | 8.34 | 0.986 |  | -2.91 | 32.32 | 0.928 |  |
| This Land is Your Land |  | -5.99 | 5.57 | 0.286 |  | -48.28 | 21.58 | 0.028 |  |
| Amazing Grace |  | 0.20 | 6.06 | 0.974 |  | 28.86 | 23.47 | 0.223 |  |
| **Virtual Live** |  | 2.89 | 6.79 | 0.672 |  | 34.47 | 26.31 | 0.194 |  |
| **In-Person Live** |  | 1.71 | 5.12 | 0.739 |  | -5.33 | 19.83 | 0.789 |  |
| Adjusted for the order of the intervention, carry over, and period. | | | | | | | | | |

| **Supplementary Table 14. Heart rate variability secondary and exploratory analyses - LF Power % compared to control** | | | | | | | | | |
| --- | --- | --- | --- | --- | --- | --- | --- | --- | --- |
|  |  | **Absolute** (during-pre) | | |  | **Relative** (100*(during-pre)/pre) | | |  |
| **Model/ Parameter** |  | **Estimate** | **Std-Err** | **P-value** |  | **Estimate** | **Std-Err** | **P-value** |  |
| **LF Power %**  **Secondary Analysis** |  |  |  |  |  |  |  |  |  |
| **Virtual Live** |  | -1.31 | 6.55 | 0.842 |  | 3.09 | 15.40 | 0.842 |  |
| **In-Person Live** |  | -3.58 | 4.94 | 0.471 |  | -13.26 | 11.62 | 0.258 |  |
| **Video** |  | 4.54 | 3.90 | 0.247 |  | 1.90 | 9.17 | 0.836 |  |
| **LF power %**  **Exploratory Analysis** |  |  |  |  |  |  |  |  |  |
| **Song** |  |  |  |  |  |  |  |  |  |
| Hey Jude |  | 3.36 | 5.73 | 0.559 |  | 7.24 | 13.49 | 0.593 |  |
| Jolene |  | -0.31 | 8.45 | 0.970 |  | -0.57 | 19.88 | 0.977 |  |
| This Land is Your Land |  | 5.14 | 5.73 | 0.372 |  | 2.19 | 13.48 | 0.871 |  |
| Amazing Grace |  | -0.94 | 6.21 | 0.880 |  | -5.14 | 14.60 | 0.726 |  |
| **Virtual Live** |  | -1.51 | 6.73 | 0.823 |  | 3.37 | 15.83 | 0.832 |  |
| **In-Person Live** |  | -3.54 | 5.05 | 0.486 |  | -13.59 | 11.88 | 0.256 |  |
| Adjusted for the order of the intervention, carry over, and period. | | | | | | | | | |

| **Supplementary Table 15. Heart rate variability secondary and exploratory analyses – LF/HF Ratio compared to control** | | | | | | | | | |
| --- | --- | --- | --- | --- | --- | --- | --- | --- | --- |
|  |  | **Absolute** (during-pre) | | |  | **Relative** (100*(during-pre)/pre) | | |  |
| **Model/ Parameter** |  | **Estimate** | **Std-Err** | **P-value** |  | **Estimate** | **Std-Err** | **P-value** |  |
| **LF/HF Ratio**  **Secondary Analysis** |  |  |  |  |  |  |  |  |  |
| **Virtual Live** |  | -2.01 | 1.73 | 0.248 |  | -79.25 | 74.66 | 0.292 |  |
| **In-Person Live** |  | 1.38 | 1.29 | 0.288 |  | 39.65 | 55.94 | 0.481 |  |
| **Video** |  | 2.82 | 1.03 | 0.008 |  | 97.82 | 44.50 | 0.031 |  |
| **LF/HF Ratio**  **Exploratory Analysis** |  |  |  |  |  |  |  |  |  |
| **Song** |  |  |  |  |  |  |  |  |  |
| Hey Jude |  | 1.15 | 1.40 | 0.413 |  | -29.01 | 61.75 | 0.640 |  |
| Jolene |  | 2.34 | 2.08 | 0.263 |  | 221.96 | 91.48 | 0.018 |  |
| This Land is Your Land |  | 4.80 | 1.41 | 0.001 |  | 104.47 | 62.30 | 0.098 |  |
| Amazing Grace |  | -2.39 | 1.52 | 0.120 |  | -5.94 | 66.92 | 0.930 |  |
| **Virtual Live** |  | -2.60 | 1.66 | 0.123 |  | -102.79 | 73.29 | 0.165 |  |
| **In-Person Live** |  | 1.64 | 1.24 | 0.191 |  | 50.69 | 54.67 | 0.357 |  |
| Adjusted for the order of the intervention, carry over, and period. | | | | | | | | | |

| **Supplementary Table 16. Heart rate variability secondary and exploratory analyses - Ln HF Power compared to control** | | | | | | | | | |
| --- | --- | --- | --- | --- | --- | --- | --- | --- | --- |
|  |  | **Absolute** (during-pre) | | |  | **Relative** (100*(during-pre)/pre) | | |  |
| **Model/ Parameter** |  | **Estimate** | **Std-Err** | **P-value** |  | **Estimate** | **Std-Err** | **P-value** |  |
| **Ln HF Power**  **Secondary Analysis** |  |  |  |  |  |  |  |  |  |
| **Virtual Live** |  | -0.12 | 0.43 | 0.774 |  | -0.18 | 8.54 | 0.983 |  |
| **In-Person Live** |  | -0.50 | 0.35 | 0.156 |  | -10.49 | 6.87 | 0.131 |  |
| **Video** |  | -0.89 | 0.29 | 0.003 |  | -15.63 | 5.79 | 0.009 |  |
| **Carry In-Person Live** |  | -0.82 | 0.40 | 0.043 |  | -17.29 | 7.96 | 0.033 |  |
| **Carry Video** |  | -1.10 | 0.39 | 0.007 |  | -21.96 | 7.85 | 0.007 |  |
| **Ln HF Power**  **Exploratory Analysis** |  |  |  |  |  |  |  |  |  |
| **Song** |  |  |  |  |  |  |  |  |  |
| Hey Jude |  | -0.64 | 0.37 | 0.086 |  | -10.77 | 7.40 | 0.150 |  |
| Jolene |  | -0.67 | 0.55 | 0.229 |  | -5.61 | 10.97 | 0.610 |  |
| This Land is Your Land |  | -0.27 | 0.37 | 0.460 |  | -7.05 | 7.39 | 0.344 |  |
| Amazing Grace |  | -0.38 | 0.40 | 0.356 |  | -6.82 | 8.08 | 0.402 |  |
| **Virtual Live** |  | -0.15 | 0.44 | 0.741 |  | -0.60 | 8.79 | 0.946 |  |
| **In-Person Live** |  | -0.47 | 0.35 | 0.181 |  | -10.29 | 7.03 | 0.147 |  |
| **Carry In-Person Live** |  | -0.81 | 0.41 | 0.052 |  | -17.54 | 8.23 | 0.036 |  |
| **Carry Video** |  | -1.10 | 0.40 | 0.008 |  | -22.06 | 8.03 | 0.008 |  |
| Adjusted for the order of the intervention, carry over, and period. Units for Ln HF Power are milliseconds squared (ms^2^). | | | | | | | | | |

| **Supplementary Table 17. Description of singing interventions** | | | | | | |
| --- | --- | --- | --- | --- | --- | --- |
|  |  | **Singing Intervention 1**  **(30 minutes)** |  |  | **Singing Intervention 2**  **(30 minutes)** |  |
|  | Format | Pre-recorded instructional sing-along video |  |  | Live, in-person singing session |  |
|  | Instructor | Associate Professor of Voice |  |  | Board-certified music therapist |  |
|  | Warm-up | 10 minutes, including SOVT & breathing exercises |  |  | 10 minutes, including SOVT & breathing exercises |  |
|  | Song choices | Choose 2 out of 4 (each 10 minutes in duration) |  |  | Choose 2 out of 40+ (each 10 minutes in duration) |  |
|  | Instructor instrument | Piano |  |  | Guitar or Keyboard |  |
|  | Other body movements | Tongue, facial exercises |  |  | Tongue |  |
|  |  |  |  |  |  |  |
